# Heat-Related Emergency Department Visits During the Northwestern Heat Wave — United States, June 2021

**DOI:** 10.15585/mmwr.mm7029e1

**Published:** 2021-07-23

**Authors:** Paul J. Schramm, Ambarish Vaidyanathan, Lakshmi Radhakrishnan, Abigail Gates, Kathleen Hartnett, Patrick Breysse

**Affiliations:** ^1^Climate and Health Program, Division of Environmental Health Science and Practice, National Center for Environmental Health, CDC; ^2^National Syndromic Surveillance Program, Division for Health Informatics and Surveillance, Center for Surveillance, Epidemiology and Laboratory Services, CDC; ^3^Office of the Director, National Center for Environmental Health, CDC.

Record high temperatures are occurring more frequently in the United States, and climate change is causing heat waves to become more intense (*1*), directly impacting human health, including heat-related illnesses and deaths. On average, approximately 700 heat-related deaths occur in the United States each year (*2*). In the northwestern United States, increasing temperatures are projected to cause significant adverse health effects in the coming years (*3*). During June 25–30, 2021, most of Oregon and Washington were under a National Weather Service excessive heat warning.* Hot conditions persisted in parts of Oregon, Washington, or Idaho through at least July 14, 2021. The record-breaking heat had the largest impact in Oregon and Washington, especially the Portland metropolitan area, with temperatures reaching 116°F (46.7°C), which is 42°F (5.6°C) hotter than the average daily maximum June temperature.

Data from the National Syndromic Surveillance Program (NSSP)^†^ were analyzed to examine patterns in heat-related illness emergency department (ED) visits during the June 2021 heat wave and the month preceding it in the northwestern United States. Heat-related ED visits were analyzed for U.S. Department of Health and Human Services (HHS) Region 10, which includes Alaska, Idaho, Oregon, and Washington, during May 1–June 30 in 2019 and 2021. ED visits were compared with those in the rest of the nation and to corresponding months in 2019; comparison data from 2019 were selected to diminish potential confounding effects of COVID-19 on ED visit trends in 2020, such as changes in health care seeking behavior. Heat-related illness ED visits were identified using a combination of free text describing the patient’s reason for visit (chief complaint) and administrative discharge diagnoses indicating exposure to high ambient temperature. To account for changes in facilities sharing data with NSSP, 2019 to 2021 comparisons were restricted to EDs with consistent reporting during the study period^§^ and at least one visit for heat-related illness. Daily counts and rates (mean number of ED visits for heat-related illnesses divided by mean total number of ED visits multiplied by 100,000) were analyzed by age group (0–17, 18–25, 26–54, 55–64, 65–74, and ≥75 years) and sex. This activity was reviewed by CDC and was conducted consistent with applicable federal law and CDC policy.^¶^

During May and June 2021, HHS Region 10 had 3,504 heat-related illness ED visits (median = seven per day [range = 0–1,090]). Approximately 79% (2,779) of these occurred during 6 days (June 25–30), when most of Oregon and Washington were under an excessive heat warning. A clear peak was detected on June 28, with 1,090 heat-related illness ED visits. After correcting for changes in reporting to facilitate comparison with 2019, the analysis found that 1,038 heat-related illness ED visits occurred on June 28, 2021 (Figure), compared with nine heat-related illness ED visits on the same date in 2019. Examining the rate of heat-related illness ED visits by age group and sex highlighted the risk associated with individual characteristics. Among the demographic groups considered, the most affected groups during June were males (862 per 100,000 ED visits) and persons aged ≥75 years (1,094 per 100,000 ED visits). Although HHS Region 10 includes approximately 4% of the U.S. population, it accounted for approximately 15% of the total heat-related illness ED visits nationwide during June. The mean daily number of heat-related illness ED visits in HHS Region 10 for June 2021 (102) was more than seven times higher than that in June 2019 (14), and during June 25–30, 2021 (424) was 69 times higher than that during the same days in 2019 (six), when no heat advisory was in effect.

**FIGURE:**
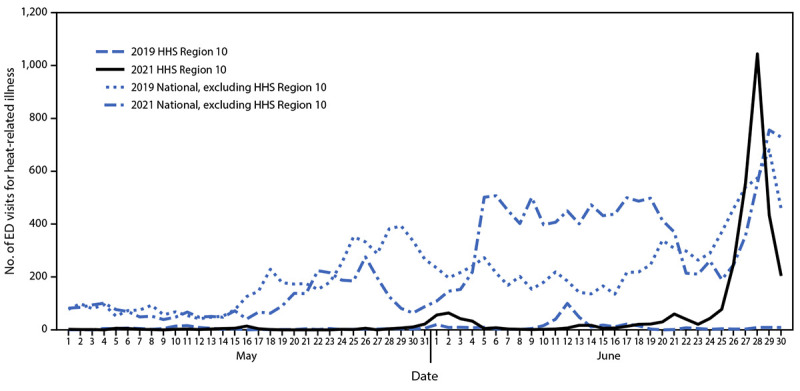
Number of emergency department visits for heat-related illness* in U.S. Department of Health and Human Services Region 10^†^ and nationwide (excluding Region 10), by year^§^ — National Syndromic Surveillance Program,^¶^ United States, May 1–June 30, 2019 and May 1–June 30, 2021 **Abbreviations:** ED = emergency department; HHS = U.S. Department of Health and Human Services; NSSP = National Syndromic Surveillance Program. * ED visits for heat-related illness were identified using a combination of free-text reason for visit (chief complaint) and discharge diagnosis codes indicating exposure to high ambient temperature. ^†^ HHS Region 10 includes Alaska, Idaho, Oregon, and Washington. ^§^ To limit the effect of data quality on trends, all 2019–2021 comparison analyses were restricted to facilities with a coefficient of variation ≤40 and weekly average informative discharge diagnosis of ≥75% throughout the analysis period May 1–June 30, 2019 and May 1–June 30, 2021, so that only facilities with consistent reporting and more complete data were included. This helps account for changes over time in the number of facilities sharing data with NSSP, which can impact analyses involving multi-year comparisons. ^¶^ NSSP is a collaboration between CDC, local and state health departments, federal agencies, health care facilities, independent clinical laboratories, and a university-affiliated research center. NSSP receives data from 71% of nonfederal EDs nationwide, although <50% of ED facilities from California, Hawaii, Iowa, Ohio, Minnesota, and Oklahoma currently participate in NSSP. Among all visit data received by NSSP, 78% are reported within 24 hours of the clinical encounter. NSSP collects ED visit information (chief complaint and administrative discharge diagnosis) and patient demographic details such as age, gender, race, and ethnicity. Diagnosis information is collected using the *International Classification of Diseases, Tenth Revision, Clinical Modification* (ICD-10-CM) and (ICD-9-CM), and Systematized Nomenclature of Medicine (SNOMED) codes.

The findings in this report are subject to at least four limitations. First, NSSP data are not nationally representative, and participation can vary by HHS Region. Second, ED visit volume can change as participating facilities are added to the system, close, or change their reporting. Third, coding practices and completeness of chief complaint text and keywords might be inconsistent. To limit the effects of these instabilities, the analysis only included facilities that consistently shared data during May 1, 2019–June 30, 2021 and those with >75% completeness in average weekly diagnosis information. Finally, these data reflect ED visits only, and do not capture persons who sought treatment elsewhere, which likely resulted in underestimation of heat-related illness prevalence.

The June 2021 northwestern heat wave had a sizeable public health impact. Health departments can develop and implement heat response plans, identify at-risk neighborhoods and populations, open cooling centers, and use data to guide public health policy and action to protect their communities from heat-related illness and deaths, especially among disproportionately affected populations. Environmental emergencies necessitate timely mechanisms for tracking health information. Syndromic surveillance helps meet this need through near real-time monitoring of health conditions to trigger response, guide policy, allocate resources, and save lives.

## Footnotes

*
https://www.weather.gov/

† NSSP is a collaboration between CDC, local and state health departments, federal agencies, health care facilities, independent clinical laboratories, and a university-affiliated research center. NSSP receives data from 71% of nonfederal emergency departments (EDs) nationwide, although <50% of ED facilities from California, Hawaii, Iowa, Ohio, Minnesota, and Oklahoma currently participate in NSSP. Among all visit data received by NSSP, 78% are reported within 24 hours of the clinical encounter. NSSP collects ED visit information (chief complaint and administrative discharge diagnosis) and patient demographic details such as age, gender, race, and ethnicity. Diagnosis information is collected using the *International Classification of Diseases, Tenth Revision, Clinical Modification* (ICD-10-CM) and (ICD-9-CM), and Systematized Nomenclature of Medicine (SNOMED) codes.

§ To limit the effect of data quality on trends, all 2019–2021 comparison analyses were restricted to facilities with a coefficient of variation ≤40 and weekly average informative discharge diagnosis of ≥75% throughout the analysis period May 1–June 30, 2019 and May 1–June 30, 2021, so that only facilities with consistent reporting and more complete data were included. This helps account for changes over time in the number of facilities sharing data with NSSP, which can impact analyses involving multi-year comparisons.

¶ 45 C.F.R. part 46, 21 C.F.R. part 56; 42 U.S.C. Sect. 241(d); 5 U.S.C. Sect. 552a; 44 U.S.C. Sect. 3501 et seq.
