# *Erratum:* Vol. 70, No. 29

**DOI:** 10.15585/mmwr.mm7032a4

**Published:** 2021-08-13

In the report, “Heat-Related Emergency Department Visits During the Northwestern Heat Wave — United States, June 2021,” on page 1020, the last sentence of the first paragraph should have read, “The record-breaking heat had the largest impact in Oregon and Washington, especially the Portland metropolitan area, with temperatures reaching 116°F (46.7°C), which is 42°F **(23.3°C)** hotter than the average daily maximum June temperature.”

